# Sodium bicarbonate cotransporter NBCn1/Slc4a7 affects locomotor activity and hearing in mice

**DOI:** 10.1016/j.bbr.2020.113065

**Published:** 2020-12-13

**Authors:** Inyeong Choi, Kristian Beedholm, Vibeke S. Dam, Seong-Ho Bae, Donald J. Noble, Sandra M. Garraway, Christian Aalkjaer, Ebbe Boedtkjer

**Affiliations:** a Department of Physiology, Emory University School of Medicine, Atlanta, USA; b Department of Biology, Aarhus University, Aarhus, Denmark; c Department of Biomedicine, Aarhus University, Aarhus, Denmark; d Department of Medicine, Division of Cardiology, Emory University School of Medicine, Atlanta, USA

**Keywords:** Sodium bicarbonate transporter, Acid base transporter, Knockout mice, Mouse behavior, Vision, Hearing

## Abstract

Despite a widespread expression pattern in the central nervous system, the role of the sodium bicarbonate cotransporter NBCn1/Slc4a7 has not been investigated for locomotor activity, emotion and cognition. Here, we addressed the behavioral consequences of NBCn1 knockout and evaluated hearing and vision that are reportedly impaired in an earlier line of NBCn1 knockout mice and may contribute to behavioral changes. In a circular open field, the knockout mice traveled a shorter distance, especially in the periphery of the chamber, than wildtype littermates. The knockout mice also traveled a shorter total distance in a home cage-like open field. Rearing and grooming behaviors were reduced. The knockout and control mice displayed similar time spent and number of open and closed arms in the elevated plus maze test, indicating negligible change in anxiety. In the Morris water maze test, both groups of mice learned the location of an escape platform within comparable time on the training trials and showed similar platform identification on the probe trial. The knockout mice maintained normal visual responses in the optokinetic drum and produced evoked potentials in response to light stimuli. However, these mice failed to produce auditory evoked potentials. qPCR revealed a robust expression of an alternatively transcribed NBCn1 variant in the knockout mouse retina. These results indicate that NBCn1 deletion leads to reduced locomotor activity in mice by affecting their exploratory behaviors or emotionality. The deletion also causes hearing loss, but its effect on vision varies between different lines of knockout mice.

## 1. Introduction

The sodium bicarbonate cotransporter NBCn1/SLC4A7 is a membrane protein that mediates electroneutral uptake of Na^+^ and ${\text{HCO}}_{3}^{-}$ into cells [1,2]. The ${\text{HCO}}_{3}^{-}$ movement *via* NBCn1 is responsible for intracellular pH (pH_i_) regulation in numerous cell types and facilitates transepithelial ${\text{HCO}}_{3}^{-}$ secretion and absorption across epithelial cells [3,4]. Genome-wide association studies identified *SLC4A7* nucleotide variations that are linked to breast cancer [5], hypertension [6], addiction [7], and environmentally toxic metal accumulation in the body [8]. Functional and physiological implication of some of these variations has been examined [9–11], and the results show their close association with changes in transporter protein expression. Conclusively, various studies demonstrate that NBCn1 plays important roles in regulating cell, tissue, and organ functions in the body.

Knockout (KO) mouse models in which the *Slc4a7* gene is disrupted have been valuable for obtaining information on physiological and pathophysiological roles of NBCn1 [12]. Three lines of NBCn1 KO mice are reported [13–15]. The first line of KO mice, which was generated by a deletion of 148 bp in exon 5, develops visual and hearing defects [13]. These mice exhibit a progressive degeneration of photoreceptor cells in the retina, resulting in a 90 % loss of the electrical activity of the retina in response to a light stimulus at about one year. The animals also develop hearing loss due to degeneration of hair cells in the organ of Corti, a core component of the cochlea, and morphological changes in the cochlear duct [16]. The second line of mice was produced by integration of a gene trap vector into the intron sequence between exon 3 and exon 4, and shows a 70 % reduction in NBCn1 protein expression [15]. The mice have normal viability but phenotypic details are not available. The third line of mice, which has been most intensively investigated and is evaluated in the current study, was produced by inactivation of the promoter site in which a gene trap vector was inserted 434 bases upstream of the MEAD start codon [14]. These mice are mildly hypertensive, which can be explained by impaired activity of nitric oxide synthase in vascular endothelial cells with the consequence of abnormal vascular tone and contractility [14,17]. The animals also fail to secrete ${\text{HCO}}_{3}^{-}$ in the duodenum [18] and display delayed build-up and decreased thickness of the adherent mucus layer in the colon [19]. In addition, they show delayed breast tumor development and decelerated tumor growth [20–22]. Overall, studies using these knockout models have advanced our understanding of the physiological and pathophysiological functions of NBCn1 in different organs and provided a foundation for possible future NBCn1-directed treatment of pathological conditions.

We recently reported that *N*-methyl-D-aspartate receptors (NMDARs) were downregulated in NBCn1 KO mice, resulting in decreased susceptibility to NMDA-mediated excitotoxicity and seizure activity [23]. NMDARs are not only important for neurotoxicity but also mediate learning and memory, cognition and reward function [24]. The receptors also play a role in controlling emotional states such as anxiety and fear [25]. These receptor properties led to the question of whether NBCn1 KO mice experience altered learning ability and emotional behaviors, especially anxiety. Furthermore, it is unclear whether these KO mice develop vision and hearing impairments as shown in the first line of KO mice. Vision and hearing defects can affect physical, emotional, and cognitive functions [26,27]. Thus, in this study, we performed a battery of tests that examine general locomotor activity, anxiety-like behavior, spatial learning, and visual and hearing function in NBCn1 KO mice. The results show that, while some of the features are similar to those in wildtype mice and other KO mouse lines, there are differences with respect to general locomotor activity and visual/auditory function.

## 2. Materials and methods

### 2.1. Mice

The experiments in this study were conducted in accordance with the National Institute of Health Guide for the Care and Use of Laboratory Animals and were approved by the Institutional Animal Care and Use Committee at Emory University and the Danish Animal Experiments Inspectorate. The generation and basic characterization of NBCn1 KO mice on C57BL/6 J background, as well as genotyping by PCR, are described previously [14]. Controls were age-matched wildtype (WT) littermates. Behavioral tests were performed with mice aged 8–12 weeks, an active stage of life. Vision and hearing tests were performed with mice aged 10–12 months. At these ages, the first line of KO mice [13] severely lost vision and hearing, thus permitting comparison of our study with the one previously reported. The experiments were performed with male mice to minimize potential variation caused by sex differences. Mice were housed on a 12-h light/dark cycle and experiments were conducted during the light phase. The same group of animals was subjected to the following sequence of behavioral tests: open field in a circular chamber, elevated plus-maze, and Morris water maze. A period of 1–2 weeks was allowed between tests. For home cage-like open fields and vision/hearing tests, different cohorts of mice were used. Mice were provided standard chow and water *ad libitum*.

### 2.2. Open field test

The open field apparatus was a circular chamber with opaque gray Plexiglas walls (diameter of 96.5 cm; height of 28 cm). A circle was inscribed 18 cm from the walls to divide the chamber into a smaller inner circle (area of ~3100 cm^2^) and an outer ring (area of ~3800 cm^2^). Mice were placed individually in the center of the chamber and allowed to freely explore for 10 min, during which their movement was recorded by an overhead tracking system TopScan (CleverSys, Reston, VA) equipped with active infrared sensors. In another set of open field tests, mice were placed in a home cage-like chamber (30.5 cm × 15.9 cm × 14 cm) and their travel distance was recorded for 30 min. Recordings were then analyzed using the tracking software Toxtrac [28]. Rearing and grooming behaviors were manually analyzed using the open-source video analysis software VirtualDub (http://www.virtualdub.org). Rearing was counted when animals stood on their hind legs, and grooming was counted when animals displayed self-directed licking or scratching. An experimenter who was blind to the animal’s genotype scored videotaped recordings.

### 2.3. Elevated plus maze test

The elevated plus maze apparatus was constructed of Plexiglas, raised 76.2 cm (30 in. above the floor, and had two open arms and two enclosed arms arranged in a plus orientation. Each arm was projected 30.5 cm from the center. Mice were placed in the center of the maze facing one of the open arms and allowed to freely explore the apparatus for 5 min, during which their movement was recorded using TopScan. Measures included *i)* time spent in open arms and closed arms and *ii*) number of entries to open and closed arms. Animals that fell off the plus maze during the test were excluded from the study.

### 2.4. Morris water maze

The Morris water maze test was performed as described previously [29]. Briefly, a water maze consisted of a circular swim arena (132 cm diameter) in an environment rich with extra maze cues for spatial reference. Mice were placed in the water maze with their paws touching the wall from four different entry positions (south, north, east, west) in water at 23 °C. An invisible escape platform was located in the same spatial location (northeast) 1 cm below the water surface. Acquisition training was 4 trials per day for 5 days, and each trial lasted a maximum of 60 s. Mice that did not reach the platform in time were manually guided to the platform and allowed to sit on it for 10 s. A probe trial was conducted on day 6, wherein the platform was removed and mice were allowed to swim for 60 s. A video camera was mounted above the swim arena and linked to TopScan.

### 2.5. Optokinetic drum

The vision in mice was tested using an optokinetic drum as previously described [30]. Briefly, a mouse was placed on a stationary platform in the center of a drum that was covered with vertical black and white stripes on the inner wall. The drum was rotated clockwise for 1 min and counterclockwise for 1 min while head tracking movements were recorded using a video camera. The experiment was done once with 1.6 mm wide stripes (0.6 cycle per degree, cpd) and repeated on two separate days with 3.2 mm wide stripes (0.3 cpd). The recording was analyzed independently by two investigators blinded for genotype. Head movement was counted when the mouse head moved a minimum of 15 degrees horizontally in the stimulus direction.

### 2.6. Visual and auditory evoked potentials

Mice were sedated by intraperitoneal injection of ketamine/xylazine mixture and wrapped in a towel with the heads free and eyes regularly moistened by saline. Two subdermal needle electrodes (27 gauge, 12 mm) were placed close to the hyoid bone and over the occipital bone with a grounding electrode placed in the tail. The electrodes were connected to a RA4PA 4-channel Medusa Preamplifier (Tucker-Davis Technologies, Gainesville, FL) and a TDT RM2 Mobile Processor sampling with a rate of 24,414 Hz and controlled by custom-built LabVIEW software. Signals were generated using a DA channel of a USB-6251 multifunction device (National Instruments, TX) triggered by an output channel of the RM2 Processor. Signals were average responses to 200 stimulations at a rate of 20 Hz for auditory stimuli and 1 Hz for visual stimuli. Visual stimuli of 10 ms duration were generated using two green diodes positioned 5 cm from the eyes. For auditory stimulation, a speaker was placed 20 cm above the mouse head and connected to a custom-built electrostatic-driven power amplifier with 30 V polarization voltage (courtesy of Lee Miller, University of Southern Denmark, and the Bioscience Electronics Workshop, Aarhus University, Denmark). Sound stimuli of 300 μs duration (3 cycles) were sinusoidal 10 kHz signals with a root mean square amplitude of 60 dB re 20 μPa, which at 10 kHz corresponds to about 35 dB sensation level for normal hearing in mice [31]. Control experiments were performed by moving either the occipital electrode (during visual stimulation) or the hyoid electrode (during auditory stimulation) to the lower back of the mouse.

### 2.7. qPCR

Retinas and inner ears were excised from mice by microdissection and lysed using a TissueLyser II (Qiagen, Denmark). Total RNA was isolated using RNeasy Mini Kit (Qiagen) according to the manufacturer’s instructions and first-strand cDNA was produced using random decamer primers (Eurofins Genomics, Germany) and Superscript IV reverse transcriptase (Thermo Fisher, Waltham, MA). A reaction without reverse transcriptase served as a negative control to detect genomic contamination. qPCR was performed using TaqMan real-time PCR assays (Thermo Fisher) with the primers specific to NBCn1 mRNA transcribed from promoters P1 (MEAD-NBCn1), P2 (MERF-NBCn1), or both (total-NBCn1). The primer sequences are available in the Supplementary Material. Reference genes were ribosomal subunit S18 and transferrin receptor TFRC (Thermo Fisher). Reactions were performed using MX3000 P (Agilent, Santa Clara, CA) at 95 °C for 5 min, and then 50 cycles at 95 °C for 10 s, 56 °C for 20 s, and 72 °C for 30 s. The cycle threshold C_T_ was determined using the MxPro qPCR software that was supplied with the instrument. The expression of NBCs relative to a geometric mean from reference genes was calculated and changes in mRNA expression were determined. The calculated NBCn1 expression levels were then normalized to the mean value for total-NBCn1 transcripts in retinas from WT mice.

### 2.8. Statistical analysis

Data were reported as mean ± standard error of the mean (SEM). Data were analyzed using unpaired, two-tailed Student’s *t*-test for comparison between groups in the open field, elevated plus maze, optokinetic drum, and visual/auditory evoked potentials. Two-way repeated measure ANOVA with Sidak *post hoc* test was used for comparison between subjects in the Morris water maze test and qPCR, and with Fisher LSD *post hoc*test for travel distance in 5 min bins. A *p* value of less than 0.05 was considered significant. Analysis was made using GraphPad Prism 7 (GraphPad; La Jolla, CA) and Microsoft Office Excel add-in Analysis ToolPak (Redmond, WA).

## 3. Results

### 3.1. NBCn1 KO mice show reduced locomotor activity in the open field

**Fig. 1.**
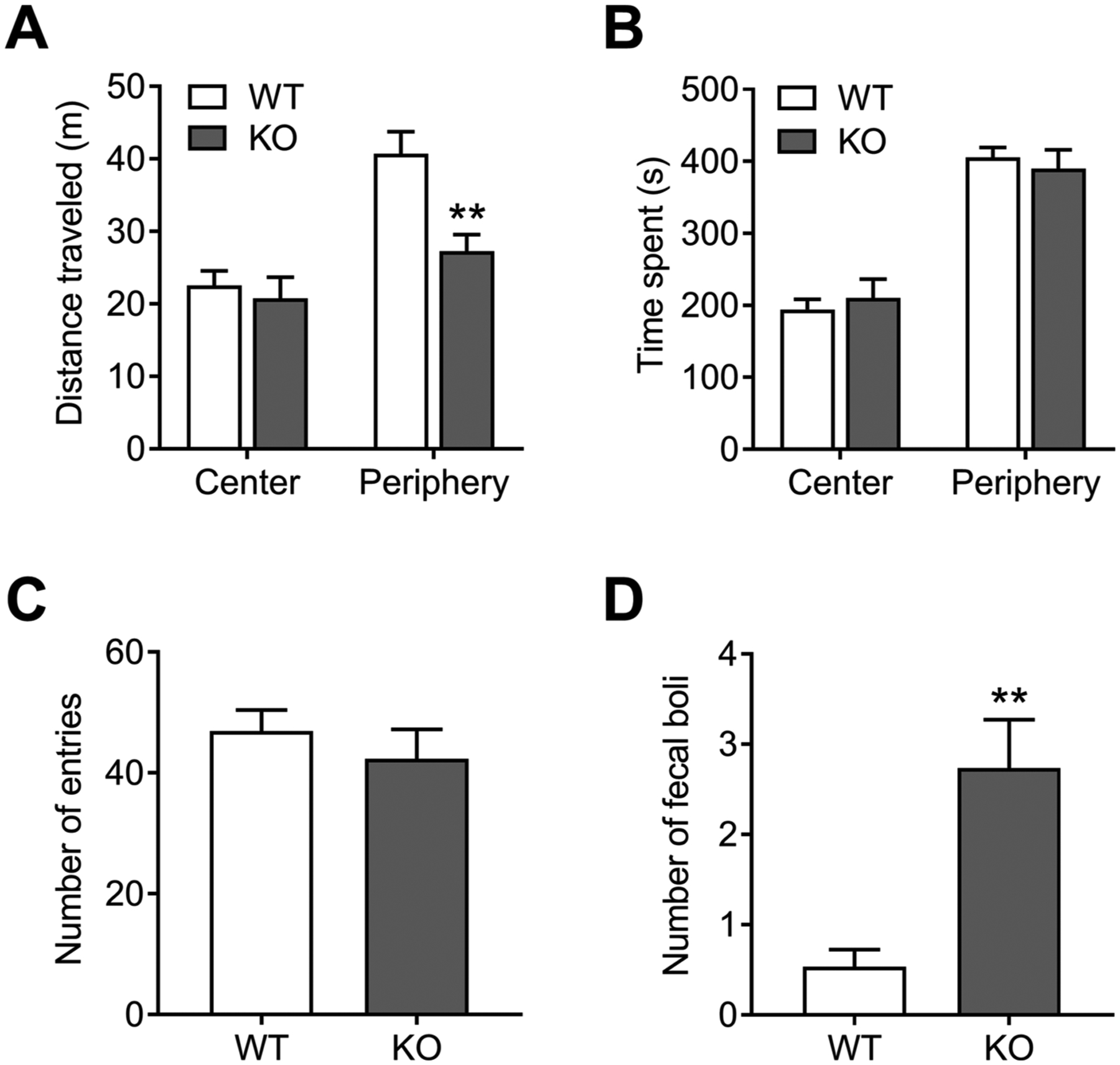
Open field test performance in NBCn1 KO mice. NBCn1 KO mice and age-matched WT littermates were individually placed in an open field chamber and their travel distance and time spent in the center and periphery of the chamber during 10 min were measured (*n* = 15/group). (**A**) Distance (in meters) traveled in the center *vs*. periphery of the chamber. (**B**) Time spent (in seconds) in the center *vs*. periphery. (**C**) Number of entries into the center. (**D**) Fecal boli deposits. **p < 0.01 compared to WT mice.

The general locomotor/anxiety-like activity in NBCn1 KO mice was tested by measuring the distance traveled in a circular open-field chamber (diameter of 96.5 cm). Fig. 1A shows the total distance traveled for 10 min in NBCn1 KO mice and WT littermates (*n* = 15/group). NBCn1 KO mice traveled a shorter total distance (62.8 ± 3.5 m for WT mice *vs*. 48.2 ± 3.9 m for KO mice; *p* = 0.01). However, this difference was observed mainly in the periphery (*p* < 0.01) of the chamber as both groups of mice traveled similar distances in the center. Both groups showed similar time spent in each region (Fig. 1B) and number of entries into the center (Fig. 1C), indicating that NBCn1 KO mice had proportionally less locomotor activity in the periphery. NBCn1 KO mice produced a higher amount of fecal boli deposits (Fig. 1D).

### 3.2. Locomotor activity and rearing/grooming behaviors are reduced in NBCn1 KO mice

**Fig. 2.**
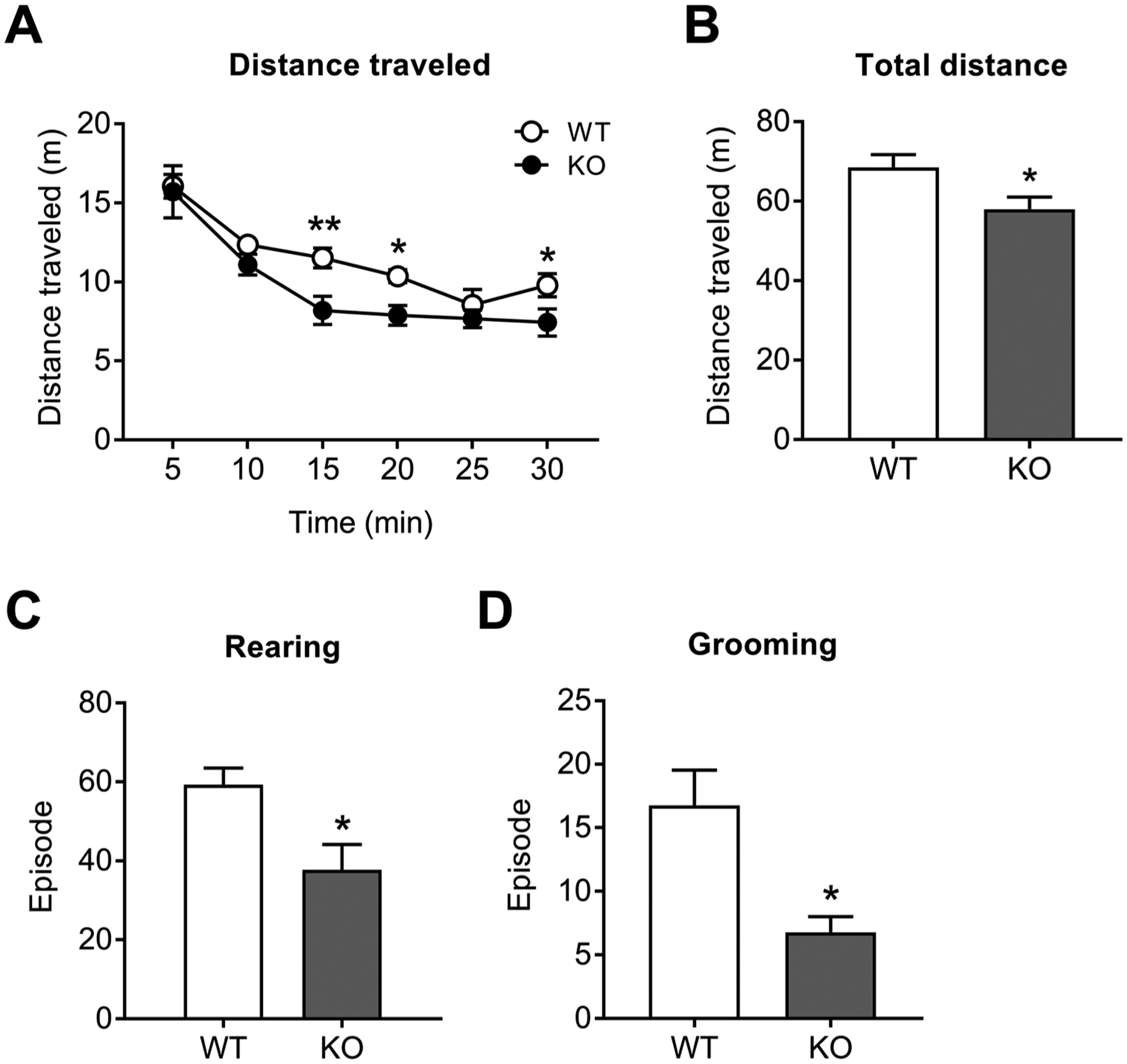
Behavioral changes in NBCn1 KO mice. Mice were individually placed in a home cage-like open field chamber and their locomotor activity and behaviors were recorded for 30 min (*n* = 8/group). (**A**) Distance traveled measured in 5-min bins (in meters). (**B**) Total distance traveled during 30 min. (**C**) Rearing episodes. (**D**) Grooming episodes. Rearing and grooming data were obtained during 10 min. *p < 0.05 and **p < 0.01 compared to WT mice.

The locomotor activity in NBCn1 KO mice was also assessed using a home cage-like open field chamber as ambulation can be affected by the chamber size and shape [32]. Fig. 2A is the distance traveled in 5 min bins for 30 min (*n* = 8/group). NBCn1 KO mice traveled less distance (*F*_1, 14_ = 5.14, two-way repeated measure ANOVA; *p* < 0.05 between genotypes). The total distance traveled was, thus, shorter in KO mice (*p* < 0.05; Student *t*-test; Fig. 2B), consistent with the results from the circular open-field chamber. NBCn1 KO mice also displayed reduced rearing and grooming behaviors (*p* < 0.05 for each; Fig. 2C & D), which are associated with exploratory activity (rearing) [33] or emotional state (rearing and grooming) [34]. The reduction was 34 % and 60 % for rearing and grooming, respectively.

### 3.3. Anxiety-related behaviors are normal in NBCn1 KO mice

**Fig. 3.**
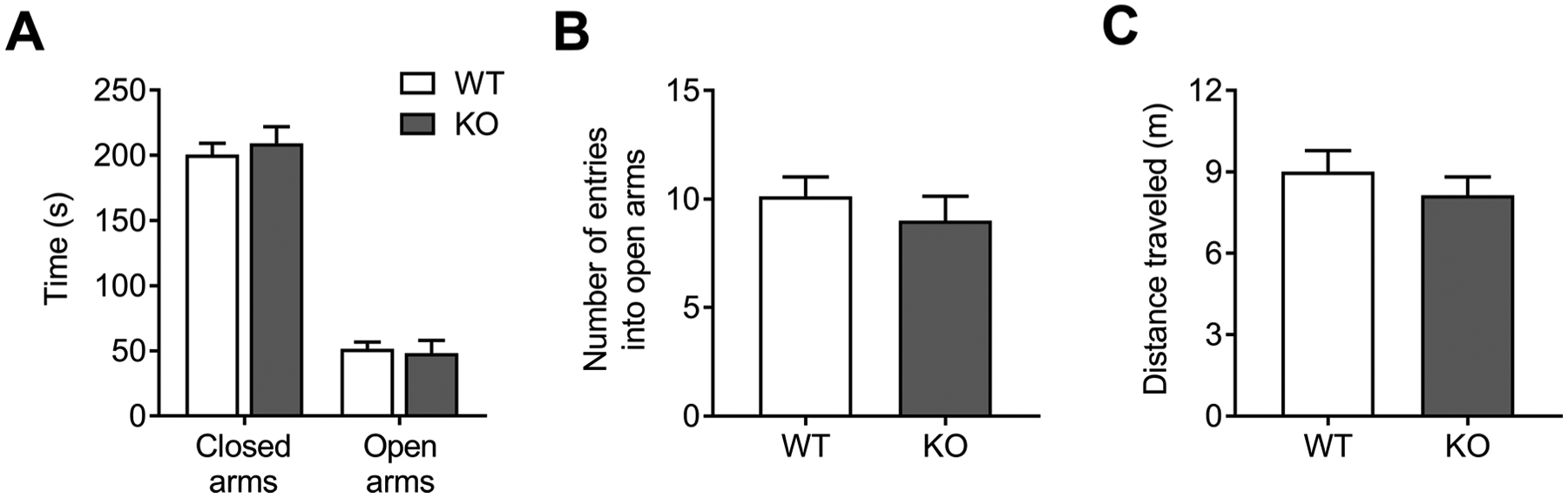
Elevated plus maze test performance in NBCn1 KO mice. (**A**) Time spent in closed arms and open arms. (**B**) Number of entries into the open arms. (**C**) Distance traveled. Data were collected from *n* = 16 WT & 15 KO mice.

In the circular open field, NBCn1 KO mice traveled normal in the center, indicating their anxiety-related behaviors are normal [35]. However, the animals produced a higher amount of fecal boli deposits, a common measure of anxiety. To address these conflicting results, we performed the elevated plus maze test. As shown in Fig. 3A, WT and KO mice spent similar amounts of time on closed arms and open arms (*p* > 0.05 for each; *n* = 16 WT and 15 KO mice). The time spent in the center was also similar between genotypes (14.2 ± 1.4 % for WT mice *vs*. 16.4 ± 2.2 % for KO mice). The number of entries into open arms was also similar (Fig. 3B). Thus, anxiety-related behaviors were normal in NBCn1 KO mice. The distance traveled was similar between genotypes (Fig. 3C), inconsistent with the result from the open field tests. We note that the elevated plus maze is specified to test anxiety and the measure of locomotor activity using this test can be variable [32,36].

### 3.4. Spatial learning and memory are normal in NBCn1 KO mice

**Fig. 4.**
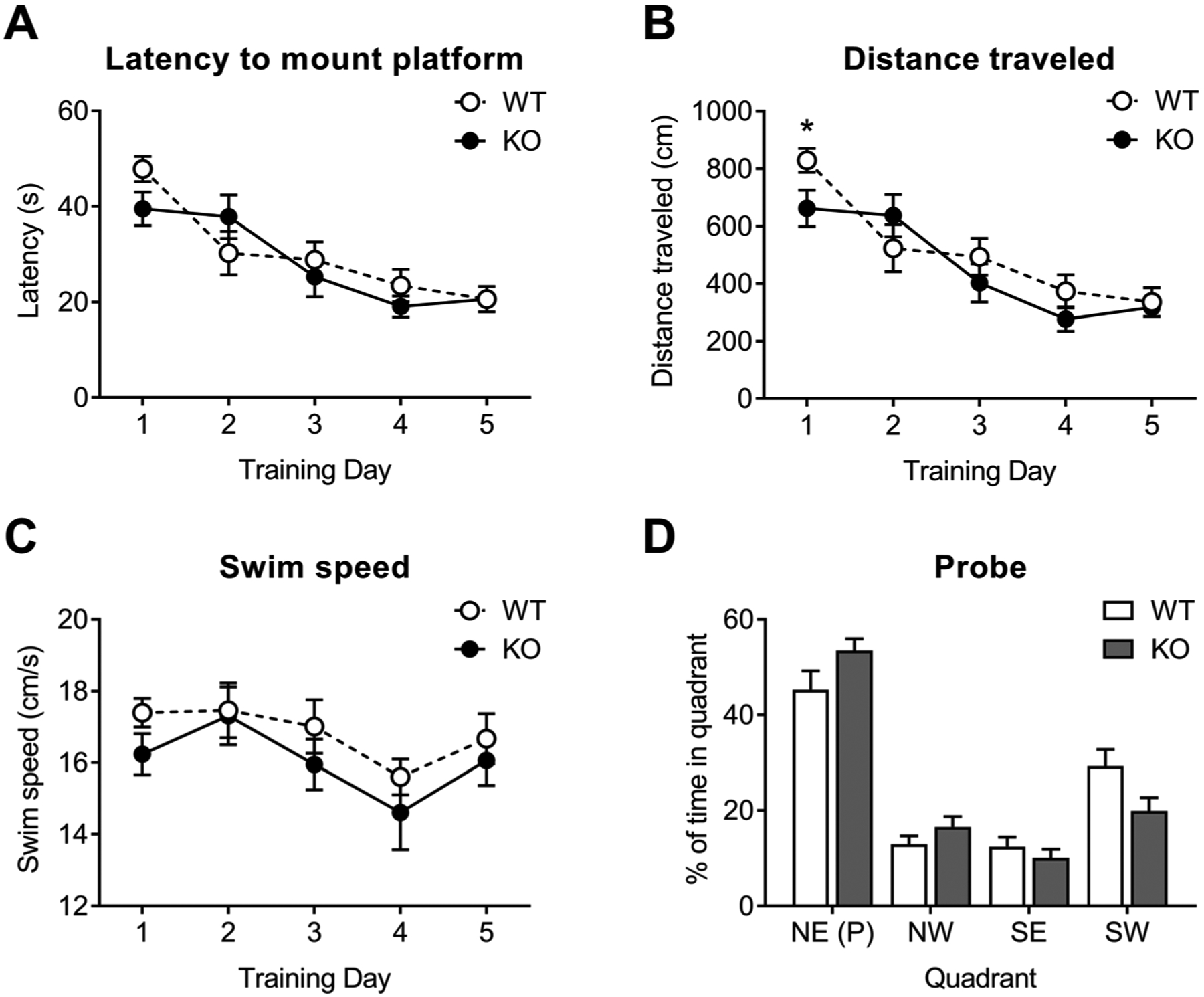
Morris water maze test performance in NBCn1 KO mice. (**A**) Escape latency to find the platform during the acquisition training for 5 days. (**B**) Distance traveled during the training. (**C**) Swim speed. (**D**) Percent of time spent in each quadrant during the probe trial on day 6. The quadrant (P) previously contained the platform during the acquisition training. NE, northeast; NW, northwest; SE, southeast; SW, southwest. Data were collected from *n* = 10/group. *p < 0.05 compared to WT mice.

Next, we examined hippocampal-based spatial learning and memory in NBCn1 KO mice using the Morris water maze. During the 5 days of training, in which animals navigated a swim arena to locate a submerged escape platform using visual cues, both WT and KO mice displayed progressively decreased latency (time to locate the platform), as shown in Fig. 4A. Two-way repeated measure ANOVA with Sidak *post hoc* test revealed no significant difference between genotypes (*F*_4,36_ = 1.22, *p* > 0.05 for genotype × latency interaction; *n* = 10/group). The distance traveled was similar between groups (Fig. 4B). NBCn1 KO mice traveled a shorter distance on the first day of training (*p* = 0.04), for which the reason is unclear. The swim speed was also similar between groups (Fig. 4C) but there was a trend toward a lower swim speed in KO mice throughout the training period. In the probe trials on day 6, both groups spent more time in the trained quadrant that previously contained the platform (Fig. 4D), thus the animals learned the platform location. Nonetheless, the time spent in the trained quadrant was similar between genotypes. These results indicate that hippocampal-based spatial learning is not altered in NBCn1 KO mice.

### 3.5. Vision is normal in NBCn1 KO mice

**Fig. 5.**
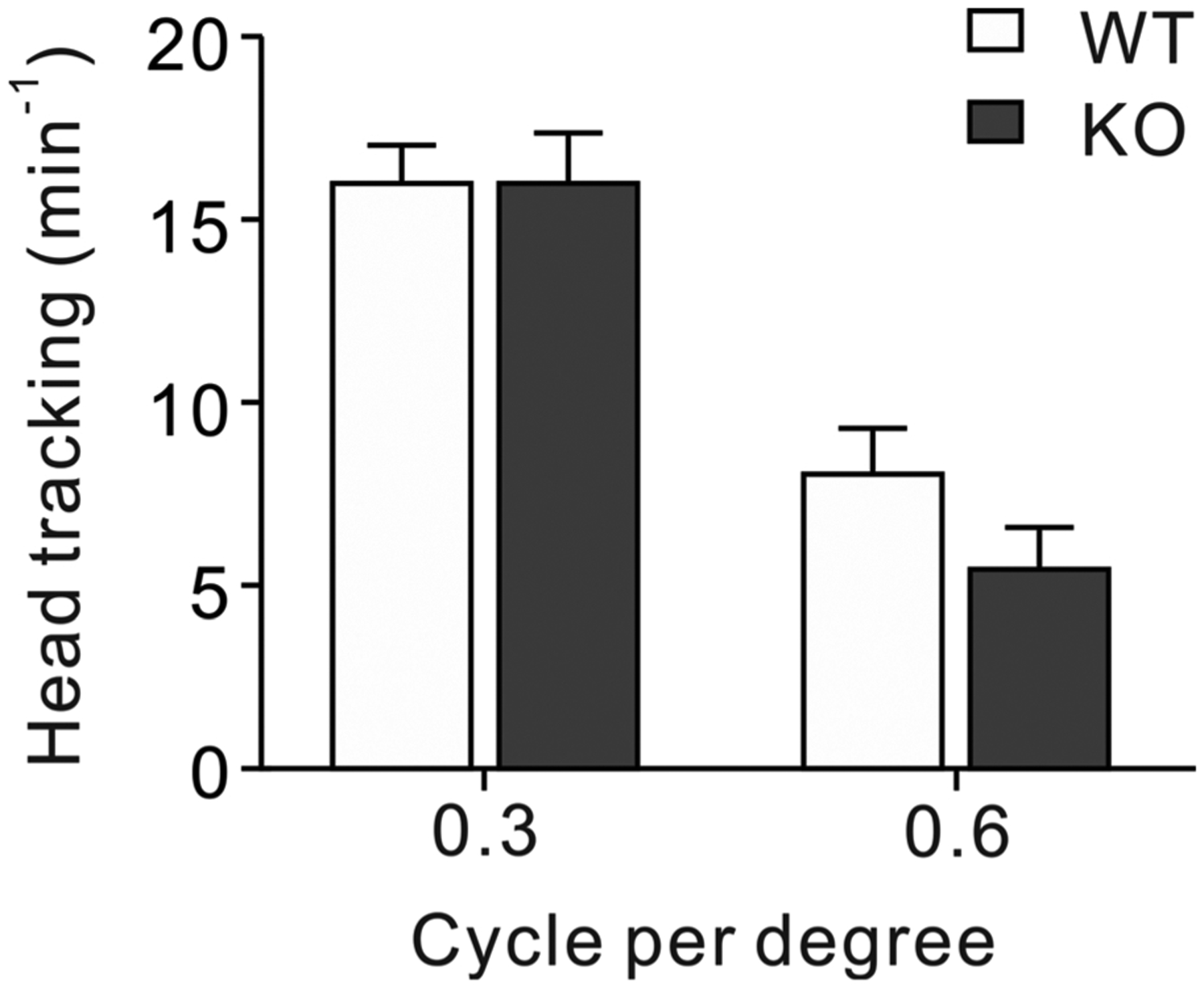
Optokinetic responses in NBCn1 KO mice. Mice at 10–12 months were placed on a stationary platform in the center of an optokinetic drum with vertical black and white stripes at a spatial frequency of 0.3 and 0.6 cycle per degree (cpd). Head tracking movement (more than 15 degrees movement of the head in the same, clockwise or anticlockwise, direction as the stripes) per minute was counted (*n* = 3–5/group).

Mice use visual cues to learn the platform location in the Morris water maze, indicating that 10–12 week-old KO mice have normal vision. This normal vision was unexpected because the first line of NBCn1 KO mice developed moderate defects in vision and hearing at these ages [13]. This disparity between our finding and the previous report led us to evaluate the vision in NBCn1 KO mice. Mice aged 10–12 months, at which the previous KO mice are reported to lose vision by 90 %, were subjected to an optokinetic response test that measures head tracking movements during the rotation of a black/white stripe-containing optokinetic drum. As shown in Fig. 5, WT and KO mice displayed similar head tracking movements at spatial frequencies of 0.3 and 0.6 cpd (*p* > 0.05 for both; *n* = 3–5). The marked decrease at 0.6 cpd is consistent with a poor performance of C57BL/6 mice to resolve a stimulus at a similar spatial frequency [37]. Importantly, similar head tracking movements between groups at both spatial frequencies indicate that visual perception in NBCn1 KO mice is equal to that in WT mice.

### 3.6. Hearing is impaired in NBCn1 KO mice

**Fig. 6.**
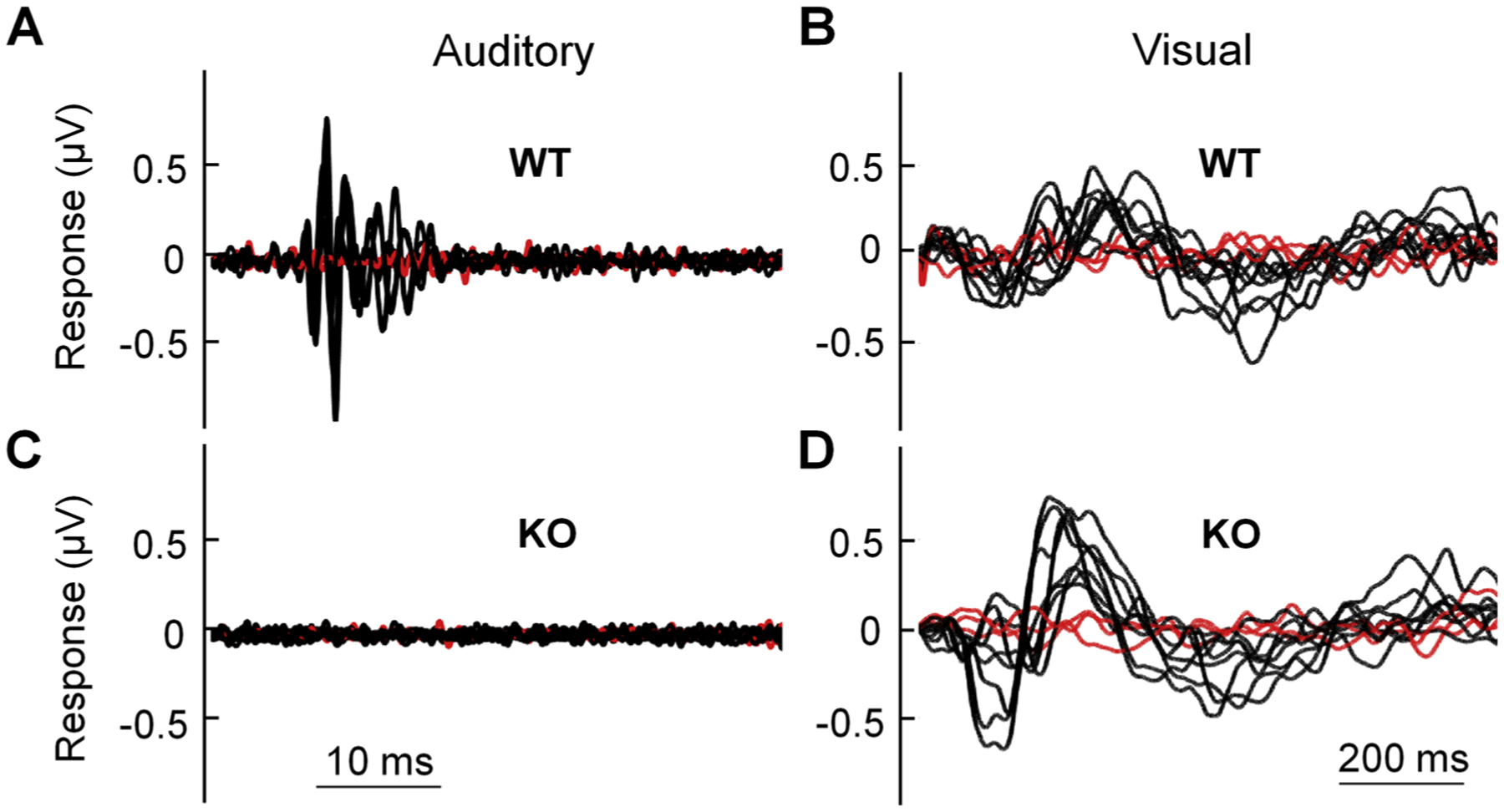
Auditory and visual responses in NBCn1 KO mice. (**A** & **C**) Auditory evoked potentials in the brainstems of WT and KO mice in response to the auditory stimuli (10 kHz signals with 60 dB sound pressure level). (**B** & **D**) Visual evoked potentials in the primary visual cortex of WT and KO mice in response to the visual stimuli (10 ms flashes of light). Traces in red are negative controls produced by moving either the hyoid electrode during auditory stimulation or the occipital electrode during visual stimulation, to the lower back of the mouse. Data were collected from *n* = 3/group.

Next, we measured visual and auditory evoked potentials (VEP and AEP, respectively) in mice. For this experiment, VEP was recorded from the primary visual cortex in response to 10 ms light flashes, and AEP from the brainstem in response to 10 kHz signals with 60 dB sound pressure level. Both WT and KO mice produced VEP to the light stimuli (*n* = 3/group; Fig. 6B & D), consistent with the results from the optokinetic drum. However, whereas WT mice produced AEP to the sound stimuli, NBCn1 KO mice produced no response (Fig. 6A & C). Control experiments by moving the recording electrodes to the lower back of the mice resulted in no response. Taken together, these results indicate that NBCn1 KO mice at the age of 10–12 months maintain normal vision but lose hearing function.

### 3.7. An alternatively transcribed NBCn1 variant is expressed in the retina of NBCn1 KO mice

**Fig. 7.**
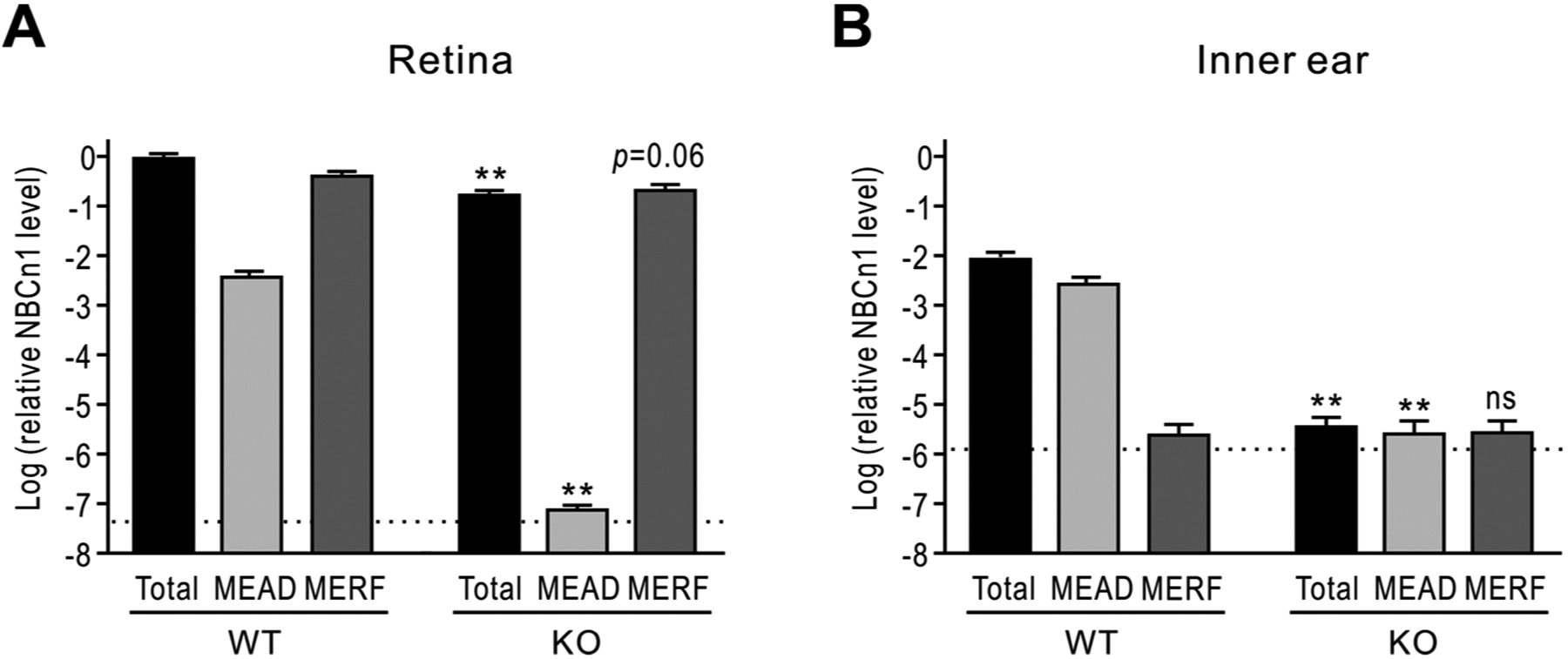
qPCR of NBCn1 variants in the mouse retina inner ear. (**A** & **B**) Expression levels of NBCn1 variants MEAD-NBCn1 and MERF-NBCn1 in mouse retina (**A**) and inner ear (**B**). qPCR was performed with three sets of primers to recognize NBCn1 mRNA produced by P1 promoter (MEAD-NBCn1), P2 promoter (MERF-NBCn1), and both (total). The C_T_ value of each NBCn1 variant was normalized to a geometric mean of reference genes 18 s RNA and TFRC using the 2^−ΔCT^ method. NBCn1 expression levels were then compared relative to the mean value for total-NBCn1 in the retina of WT mice. The dotted lines are the detection limit. Data were collected from *n* = 4 WT and 3 KO mice. **p < 0.01 compared to WT mice. ns: not significantly different.

The *Slc4a7* gene encoding NBCn1 contains promoter P1 (responsible for MEAD variant of NBCn1) located upstream of promoter P2 (responsible for MERF variant) [38]. Because NBCn1 KO mice in this study were generated by a gene trap vector insertion in P1, it is possible that the difference in vision and hearing functions could be due to different expressions of the two NBCn1 variants in the eye and ear. To address this possibility, we performed qPCR of NBCn1 in the retina and inner ear with three sets of primers that recognize MEAD-NBCn1, MERF-NBCn1, and both. As shown in Fig. 7A, MEAD-NBCn1 and MERF-NBCn1 were abundant in the retina of WT mice, but only MERF-NBCn1 was in the corresponding tissue of KO mice. Two-way repeated measure ANOVA revealed *p* < 0.01 for genotype × expression interaction (*n* = 4/group). In the inner ear of WT mice, MEAD-NBCn1 was abundant but MERF-NBCn1 was negligible (Fig. 7B). Thus, the gene knockout resulted in a basal level of NBCn1 expression in the inner ear of KO mice. Conclusively, the different expression of NBCn1 variants in the retina and inner ear correlates with the different sensory activities in vision and hearing between WT and KO mice.

## 4. Discussion

In this study, we found that NBCn1 KO mice have unequal locomotor activities in the center *vs*. periphery of the circular open field chamber. The unequal locomotor activities in different areas suggest that the motor coordination is not altered. NBCn1 is found at the neuromuscular junction [39], but its role in neurotransmission and muscle contraction is presently unclear. The previous line of NBCn1 KO mice at 1–12 months showed no apparent abnormalities in vestibular and motor function during rotarod and tilt-table experiments (stated in the discussion of the report by Bok et al. [13]). Thus, it is likely that NBCn1 plays a minor role in motor function. We think that reduced locomotor activity in our KO mice is probably due to changes in exploratory behaviors or emotionality, which are important dependent parameters in the open field test and can influence locomotor activity in rodents [40]. NBCn1 KO mice exhibit reduced rearing behaviors, which are considered exploratory activity or anxiety-related behavior [32,41,42]. Exploratory behaviors involve sensory cues such as tactile, olfactory, visual, and auditory cues to develop a spatial representation of the environment in the brain [43]. Changes in sensory perception thus affect animals’ capability to explore their environments for survival and adaptation; for example, mice with severe hearing loss display rearing deficits [44]. Given such involvement of sensory perception, we envision that the hearing defect in NBCn1 KO mice is responsible for reducing exploratory behaviors. In addition, rodent locomotor activity is also affected by emotionality such as anxiety, fear, and depression [40]. NBCn1 KO mice had reduced grooming activities, a complex repetitive and self-directed behavior that is considered as an indirect index of several emotional phenomena [34]. NBCn1 KO mice had normal behaviors in the elevated plus maze (Fig. 3); thus, anxiety is not the factor that reduces locomotor activity in these mice. The number of fecal boli in the open field was different between genotypes, but the validity of fecal boli as a measure of anxiety has been questioned because defecation can be altered by unrelated factors such as food intake [45], and we know that intestinal function is abnormal in NBCn1 KO mice [18,19]. The mechanism that underlies such reduced behaviors in NBCn1 KO mice is unclear. Given the current lack of information on pH involvement in mouse behavioral activity, it is difficult to discuss a mechanistic view on our study. We recently demonstrated that intracellular pH is low and membrane excitability is depressed in neurons from NBCn1 KO mice [46]. Thus, abnormal pH and excitability in neurons associated with exploratory behaviors or emotional activity may play a role.

NBCn1 KO mice maintain normal spatial learning behaviors in the water maze test. This result was unexpected because these mice developed marked downregulation of the NMDAR subunit GluN1 and the postsynaptic density protein PSD-95 in the hippocampus [23]. While the water maze test can be affected by factors other than spatial memory [47], we also note that there is controversy over whether NMDARs underlie spatial memory acquisition in the water maze test. In the early report [48], mice with the GluN1 deletion restricted to the hippocampal CA1 region showed impaired spatial memory in the water maze test. However, a later study using mice with the GluN1 deletion in dentate gyrus and pyramidal neurons including CA1 neurons revealed normal spatial memory [49]. Furthermore, mice with GluN1 knockout in CA3 neurons were impaired in the early stage of learning, when animals located an escape platform based on single-trial memory, but not the later stage, when animals relied on trial-independent memory [50]. Thus, NMDAR-mediated spatial memory depends on trials in the water maze, such that NMDARs are required in rapidly learning a novel platform location but not for the learning that relies on a previously experienced location [24]. Consistent with this view, there was negligible change in latency to locate and mount the platform for the first 2 training days but then KO mice improved the performance for the remaining training days (Fig. 4A). The animals subsequently learned the platform location, similar to WT mice. Overall, our results are in agreement with the current understanding that NMDARs are not always essential for all forms of hippocampus-based learning [51].

NBCn1 KO mice at 10–12 months lost hearing but maintained normal vision, different from severe losses of both vision and hearing in the first line of KO mice [13]. Both lines of KO mice have similar genetic backgrounds as they were generated by injecting 129/sv or 129/sv-derived ES cells into C57BL/6 blastocysts followed by cross breeding with C57BL/6 mice. Thus, the difference in vision and hearing between the two lines is not related to genetic background. The qPCR results (Fig. 7) demonstrate that this difference is due to alternative transcription activities of P1 and P2 promoters in the retina and inner ear. Both P1 and P2 are highly active in the retina, whereas only P1 is active in the inner ear. NBCn1 KO mice are a selective knockout of P1 activity; thus, P2 activity is intact in the retina and normal vision is maintained. We previously analyzed alternative transcriptions of human SCL4A7 gene using a transcription database and found that P1 is responsible for the majority of NBCn1 in the brain while P2 and another internal promoter are also active [9]. P2 activity in the KO mouse retina shows an example of this alternative transcription that allows the production from the same gene with different 5′ untranslated and protein-coding regions. We think that these variations would impact the translational efficiency of the NBCn1 transcript or contribute to the transcriptome and/or proteome diversity. Despite this importance, alternative transcription of P1 and P2 implies that there is a limit on the current NBCn1 KO mouse model. There should be other places, in addition to retinas, where P2 activity plays a significant role but is phenotypically undetected from the current KO mice.

In testing mouse vision and hearing, we used NBCn1 KO mice aged 10–12 months for fair comparison of our study with the one previously reported. The previous line of KO mice developed moderate defects in vision and hearing at younger ages, determined by the amplitude of maximum saturated b-wave in rod-mediated electroretinogram and the amplitude of auditory brainstem response [13]. We did not examine vision and hearing in our KO mice at < 10 months in this study; nonetheless, we think these mice develop similar hearing defects at these ages as both lines of KO mice lack NBCn1 expression in their inner ear. It will be interesting to examine age-dependent hearing defects in the current KO mice in future studies. The normal vision in NBCn1 KO mice recalls a discussion about NBCn1 involvement in sensory disorders. NBCn1 KO mice have been suggested as an animal model for type 2 Usher syndrome in humans [16,52]. However, the genomic association of *SLC4A7* with type 2 Usher phenotype was later retracted [53], and no *SLC4A7* mutation was found in Usher patients [54]. Thus, it is unlikely that NBCn1 is a candidate gene for Usher syndrome. Nonetheless, most KO mice lacking Usher proteins do not possess severe mutant phenotypes in their retinas [55]; thus, we do not completely exclude a possibility of NBCn1 contribution to this disorder.

In summary, our study shows that NBCn1 is involved in maintaining locomotor activity, exploratory behavior, and hearing function. Normal vision in NBCn1 KO mice contrasts with the early report of selective loss of photoreceptor cells and blindness caused by Slc4a7 gene knockout. The negligible change in spatial learning is intriguing, considering that these mice develop NMDAR downregulation. It will be interesting to test whether a selective and complete deletion of NBCn1 in the hippocampus leads to learning and memory defects.

## Supplementary Material

Supplementary material
