# Supplementary material for: Sodium bicarbonate cotransporter NBCn1/Slc4a7 affects locomotor activity and hearing in mice
Source: Behav Brain Res. Author manuscript; Available in PMC 2022 May 5. (PMC9069564; doi:10.1016/j.bbr.2020.113065)
Supplement: Supplementary material [file NIHMS1658597-supplement-Supplementary_material.pdf]

Table S1

Primer	Sequence
MEAD-NBCn1	Forward: CGAGCAGATGAGACCGCT
	Reverse: ACGCCGACGACTCTCTTTAC
	Matching: CAGATGAAGAAGCTGTTGTGGATC
MERF-NBCn1	Forward: GAAAGATTTCAGCTGGCGAG
	Reverse: ACGCCGACGACTCTCTTTAC
	Matching: CAGATGAAGAAGCTGTTGTGGATC
total-NBCn1	Forward: ACAGAAGGCAGAATAAGTGCAATAGA
	Reverse: AGGTTGCCCAGCAAACAATG
	Matching: AGGCAATCCCAGTTAATGATGCTCCAAAA
